# Time-dependent variations in BK polyomavirus genome from kidney transplant recipients with persistent viremia

**DOI:** 10.1038/s41598-023-40714-4

**Published:** 2023-08-19

**Authors:** Olga Mineeva-Sangwo, Elisabet Van Loon, Graciela Andrei, Dirk Kuypers, Maarten Naesens, Robert Snoeck

**Affiliations:** 1 Laboratory of Virology and Chemotherapy, Rega Institute, Department of Microbiology, Immunology and Transplantation, KU Leuven, 3000 Leuven, Belgium; 2 Nephrology and Renal Transplantation Research Group, Department of Microbiology, Immunology and Transplantation, KU Leuven, 3000 Leuven, Belgium; 3 Department of Nephrology and Renal Transplantation, University Hospitals Leuven, 3000 Leuven, Belgium

**Keywords:** Viral pathogenesis, Kidney diseases

## Abstract

BK polyomavirus (BKPyV) is a human DNA virus that resides latent in the host’s renal tissue. Reactivation occurs occasionally and in case of kidney transplantation, it can lead to polyomavirus-associated nephropathy (PVAN). Due to the lack of specific antivirals for BKPyV and despite the risk of allograft rejection, reduction of immunosuppression remains the main approach for treating PVAN. Current data suggests that mutations can accumulate over time in the major capsid protein VP1 and can lead to neutralization escape in kidney transplant recipients. Herein, we show that mutations occur throughout the entire BKPyV genome, including in VP1. Changes were identified by per-patient comparison of viral genome sequences obtained in samples from 32 kidney recipients with persistent viremia collected at different post-transplant time-points. Amino acid changes were observed in both earlier and later post-transplant samples, although some of them were only found in later samples. Changes in VP1 mainly consisted in the introduction of a new amino acid. A switch back to the conservative amino acid was also observed. This should be considered in future approaches for treating BKPyV infection in kidney transplant recipients.

## Introduction

Kidney transplantation is a treatment option for patients with end-stage renal disease and is associated with a lower risk of mortality and a better quality of life as compared to patients on dialysis^1,2^. Polyomavirus-associated nephropathy (PVAN) may lead to kidney allograft failure in renal transplant recipients^3^ and is mainly associated with the replication of BK polyomavirus (BKPyV)^4^. PVAN cases associated with JC polyomavirus (JCPyV) have also been reported^5,6^.

BKPyV is widespread in humans, with a seroprevalence ranging from 50 to 90%^7^. BKPyV can be detected in urine from both healthy and immunocompromised individuals^8,9^. In kidney transplant recipients, BKPyV replication can progress to viremia, with higher viral loads associating with higher rate of allograft biopsy-proven PVAN^10–12^. Regular screening for BKPyV viremia, especially during the first year after transplantation, is recommended to identify patients who may require treatment^4^. Treatment of BKPyV viremia and PVAN in kidney transplant recipients mainly relies on gradual reduction of immunosuppression^4^. In addition, fluoroquinolones, leflunomide, intravenous immunoglobulins, and cidofovir have been considered as adjunctive therapy, although clinical efficacy against BKPyV has not been fully established^13^. It has been reported that not all patients respond equally to treatment, and some may still show signs of BKPyV infection after intervention^14–16^.

BKPyV is a small, non-enveloped virus with a circular, double-stranded DNA genome. BKPyV genome can be broadly divided into three functional regions: a non-coding control region (NCCR), an early and a late coding region. The NCCR contains the origin of replication, promoter, and enhancer elements^17^. The early region encodes the regulatory proteins, sTag and LTag, while the late region is responsible for the encoding of the non-structural Agno protein, the major capsid protein VP1, and the minor capsid proteins VP2 and VP3^18^.

BKPyV is generally classified based on the architecture of the NCCR and the polymorphism in the VP1 gene. Archetypal NCCR has a linear O-P-Q-R-S structure while rearranged NCCR is characterized by complete or partial deletion, and/or duplication of NCCR sequence block(s)^17^. BKPyV with archetypal NCCR has been found in urine of healthy individuals^19^. BKPyV with rearranged NCCR has been observed in urine of HIV-positive patients, transplant recipients, as well as in cerebrospinal fluid of neurological patients^20–24^. In kidney transplant recipients, BKPyV with rearranged NCCR has been associated with higher plasma viral load and higher risk of histologically confirmed PVAN^20^.

Based on the VP1 gene polymorphism, BKPyV are divided into four subtypes/genotypes: I, II, III, and IV^25^. Subtypes I and IV are further divided into subgroups Ia, Ib1, Ib2, Ic, IVa1, IVa2, IVb1, IVb2, IVc1, and IVc2^26^. Subtypes/subgroups Ib1, Ib2, II, III, and IVc2 can be found in European populations^27^. In vitro studies have shown that polymorphism in VP1 can influence the growth efficiency and virulence of the virus^28–32^. Additionally, recent studies have shown that the accumulation of mutations over time in the VP1 apical surface loops can facilitate viral escape from antibody-mediated neutralization in kidney transplant recipients^33,34^.

In the present study, we aim at better understanding changes over time in the BKPyV genome in kidney transplant recipients. Building upon our previous analysis of BKPyV (and JCPyV) genome in three-month post-transplant samples from kidney recipients with viremia^35^, we characterized viral genomes from patients with persisting viremia over a longer period. We compared the viral genomes pairs of each patient and identified the changes that occurred over time, also in relation to cidofovir.

## Materials and methods

### Study cohort and sample collection

The study enrolled 85 kidney transplant recipients with viremia (BKPyV/JCPyV quantitative PCR; quantification range 2.7–7.7 log10 copies/mL) from our previous retrospective cohort study^35^. Samples were taken from patients with at least one qPCR positive plasma sample (≥ 2.7 log_10_ copies/mL) detected at 250 days or more after the patient’s first positive plasma event (48 patients). The study was approved by the Ethics Committee of the University Hospitals Leuven (S53364 and S64904) and all patients have given their written informed consent.

### Immunosuppressive and antiviral therapy

Standard immunosuppressive therapy consisted of tacrolimus, mycophenolate-mofetil, and corticosteroids. In patients with increased immunological risk, treatment also included basiliximab. Treatment of patients with BKPyV/JCPyV viremia included dose reduction of mycophenolate-mofetil. Treatment of patients with biopsy-proven PVAN consisted of the immediate withdrawal of mycophenolate-mofetil, dose reduction of tacrolimus and introduction of cidofovir.

### DNA isolation, viral genome amplification and sequencing

Total DNA was isolated from samples using QIAamp DNA Blood Midi Kit (Qiagen, Benelux BV) according to the manufacturer’s protocol. The initial sample volume was 600 µL. DNA was eluted with 150 µL of elution buffer.

Viral genome was amplified using Invitrogen SuperFi PCR Master Mix (Thermo Fisher Scientific, Brussel, Belgium) with two set of primers. The primers were designed using two sequences with GenBank access numbers V011008.1 (BKPyV) and NC_001699.1 (JCPyV). The primer sequences can be found in Supplementary Table S1.

Viral genome was sequenced using the Miseq v.2 system with paired-end (2 × 150 bp) workflow (Illumina). First, the amplicons were quantified with a Qubit fluorometer (Thermo Fisher Scientific). Next, a library was prepared using 1 ng DNA of each sample with Nextera XT kits (Illumina). The libraries were quantified and then pooled at 2nM. PhiX Control v3 (Illumina) was added to the pooled library at 5% of the total volume.

BKPyV complete genome consensus sequences were generated using the recently developed BKAnaLite bioinformatics pipeline^35^ and submitted into GenBank under accession numbers OQ230834–OQ230874 (n = 41). The JCPyV genome sequence was obtained using the CLC Genomics Workbench v12.0.3 software package (Qiagen Benelux, The Netherlands). First, the quality of the reads was checked using the QC for Sequencing Reads tool. The reads were then trimmed using the Trim Reads tool. The trimmed reads were mapped against the reference sequence (NC_001699.1) to obtain a consensus sequence. The JCPyV NCCR sequence was obtained using the Sanger method. The NCCR region was first amplified using two primers (see Supplementary Table S1) and the amplicons then sequenced and analyzed using the Big Dye Terminator v3.1 sequencing kit and ABI Prism 3730XL DNA Analyzer (Thermo Fisher Scientific). The JCPyV NCCR consensus sequence was generated using the SeqScape v2.7 software (Applied Biosystems, Foster city, CA, USA). JCPyV complete genome consensus sequences (n = 12) were submitted into GenBank under accession numbers OQ230875–OQ230886.

### BKPyV and JCPyV genotyping

BKPyV was genotyped based on the VP1 gene using an automated BKTyper v0.2 tool^36^. The BKPyV NCCR structure was defined using the same tool. In addition, NCCR sequences were compared using the multiple sequence alignment method (MAFFT v7.471) by applying default parameters^37^.

JCPyV genotyping was based on the analysis of complete JCPyV genome consensus sequences (minus the NCCR region). Sequences of various JCPyV genotypes/subtypes extracted from Genbank were included in the analysis (see Supplementary Table S2). The sequences were aligned using MAFFT. The tree was generated by the IQ-tree web service using the Maximum likelihood method^38,39^ and an appropriate substitution model was estimated using the ModelFinder method^40^ provided by the same web service. Confidence was evaluated with the UFBoot tool using the bootstrap method (1000 replicates)^41^. The obtained tree was visualized using the MEGA X application^42^. The JCPyV genotype was defined as the shortest branch distance to one reference.

JCPyV NCCR structure was determined by alignment with the archetypal JCPyV NCCR sequence (Genbank accession number M35834.1) using MAFFT.

### Coding DNA sequences mutation analysis

The analysis was performed using viral protein-coding DNA sequences. The sequences were aligned using MAFFT. The numbering of nucleotides and amino acids follows that of V01108.1 (for BKPyV) and NC_001699.1 (for JCPyV).

## Results

### Study cohort and sample collection

**Figure 1:**
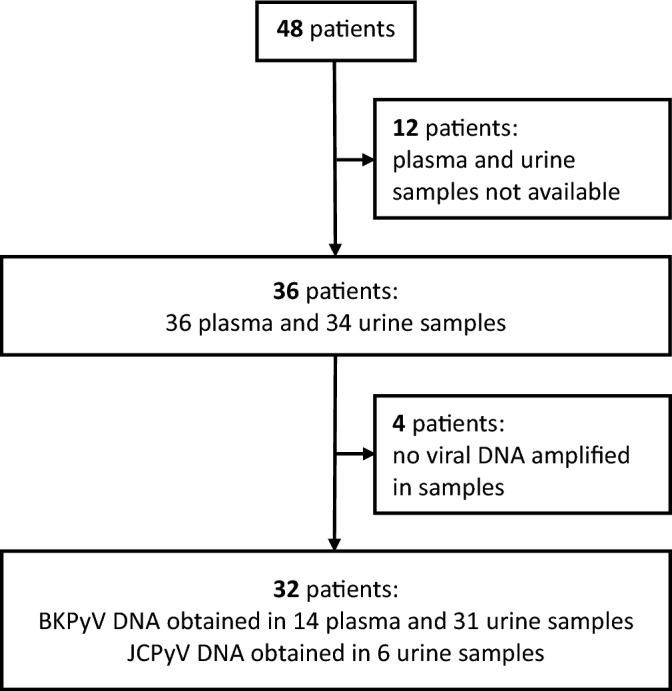
Study sample collection profile.

We analyzed BKPyV (and JCPyV) genome in samples from 48 kidney transplant recipients with persistent viremia. We were able to obtain BKPyV (whole or partial) genome consensus sequences in 14 plasma and 31 urine samples from 32 patients. With regard to JCPyV, 6 viral sequences (whole genome) were obtained (Fig. 1). The samples were collected between 339 and 427 days post-transplant for 21 patients, 709 and 830 days for 10 patients and 1479 days for one patient.

**Figure 2:**
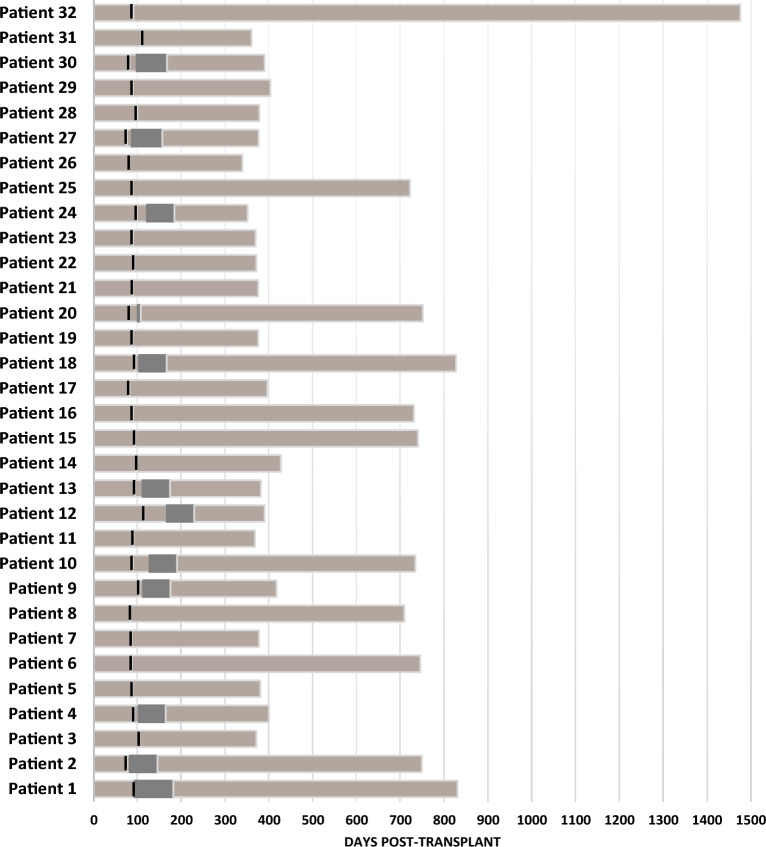
Graphical representation of post-transplant timeline for 32 patients**.** Each bar represents one patient. Black vertical line represents T1 and the furthest point on the timeline, T2. Darker color indicates a period when patient was treated with cidofovir.

We first characterized viral sequences based on the VP1 gene (BKPyV subtype/subgroup), the NCCR and the entire protein-coding region. After, we compared viral sequences with those previously obtained in three-month post-transplant samples from the same patients^35^. For BKPyV, we had 30 (whole or partial) genome consensus sequences obtained in urine samples and 21 obtained in plasma samples available for comparison. For easier identification purposes, the three-month samples are further referred to as T1, while the samples collected later are referred to as T2. For JCPyV, we had 6 (whole) genome consensus sequences obtained in T1 urine samples available for comparison. Figure 2 gives a graphical representation of a post-transplant timeline with T1 and T2 for 32 patients. Patients who received cidofovir between T1 and T2, are also highlighted in the graph (n = 12). The treatment with cidofovir lasted from 62 to 88 days for 11 patients and 7 days for one patient (Patient 20).

### BKPyV and JCPyV per-patient comparison in urine and plasma samples

Per-patient comparison of BKPyV subtype/subgroup, NCCR and entire coding region was possible for 26 paired T1-T2 urine samples and 8 pairs of plasma samples. In case of partial viral sequences, only the BKPyV subtype/subgroup or NCCR were compared. In two more pairs of urine samples and another three pairs of plasma samples, only the subtype/subgroup was compared. Comparison of NCCR was possible in one more pair of urine samples.

In total, 12 JCPyV genome sequences (6 obtained in T1 urine samples and 6 in T2 urine samples) were available for the analysis. Per-patient comparison was possible for 3 paired T1-T2 urine samples.

#### BKPyV subtype/subgroup was the same in all paired plasma samples and in all but one paired urine samples

**Figure 3:**
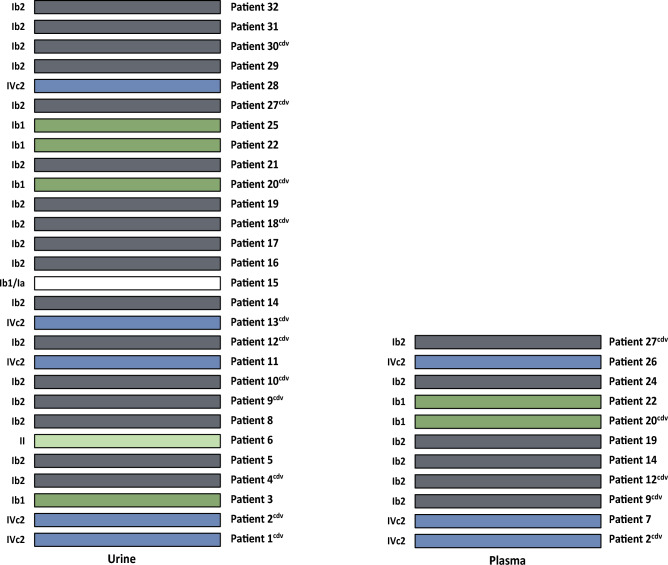
Visual representation of BKPyV subtypes/subgroups distribution in urine samples from 28 kidney recipients (left) and plasma samples from 11 kidney recipients (right) collected at T1 and T2. Each bar shows BKPyV subtypes/subgroups obtained at T1 and T2 for one patient. Colored bars indicate that the same BKPyV subtype/subgroup was found in the patient at both T1 and T2. cdv, cidofovir.

Comparison of urine samples per patient showed that most of the patients (n = 27; 96%) had the same BKPyV subtype/subgroup at T1 and T2. Subgroup Ib2 was found in most cases (n = 17; 60.7%), followed by subtype IVc2 (n = 5; 17.8%), and Ib1 (n = 4; 14.2%). One patient had subgroup II in urine samples. Another patient had subgroup Ib1 in urine sample at T1 and subgroup Ia at T2 (Fig. 3). The BKPyV subtype/subgroup was the same in plasma samples at both T1 and T2 for each patient. Subtype Ib2 was found in 6 patients (54.5%), IVc2 in 3 patients (27.2%), and Ib1 in 2 patients (18.1%) (Fig. 3).

#### Changes in BKPyV NCCR were detected in one fifth of paired urine samples and in half of paired plasma samples

**Figure 4:**
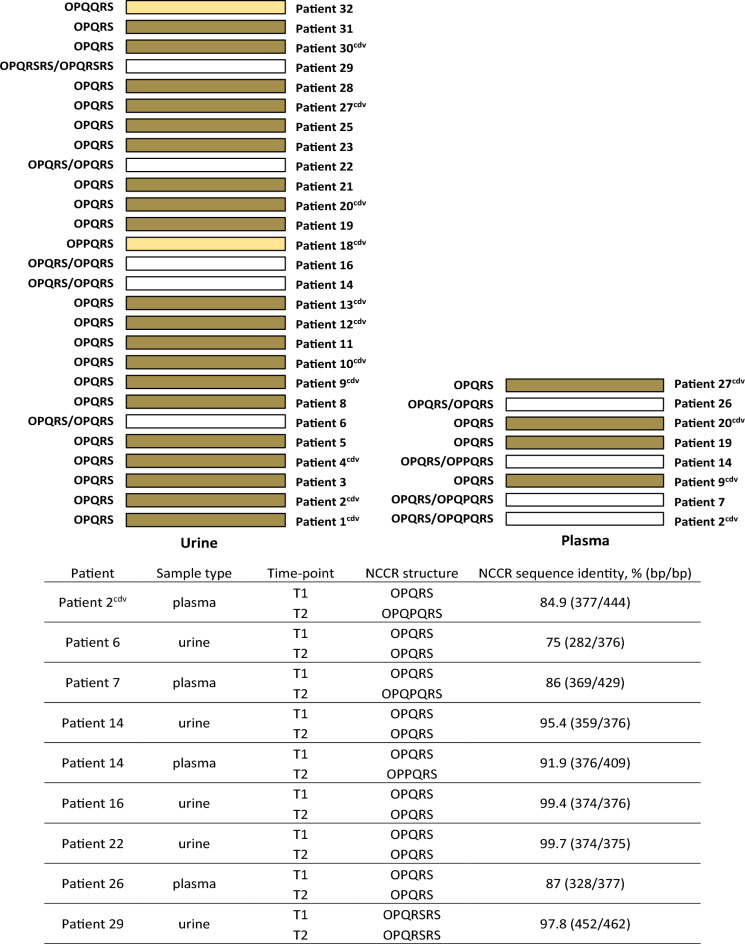
Upper panel: visual representation of genetic changes observed in BKPyV NCCR sequences obtained in urine and plasma samples at T1 and T2 from 27 and 8 kidney recipients, respectively. Lower panel: overview of patients who had NCCR changes in urine and/or plasma samples. Each bar shows NCCR sequences obtained at T1 and T2 for one patient. Colored bars indicate that the NCCR sequences were identical at both T1 and T2 for the patient. cdv, cidofovir, bp, base pairs.

Analysis of NCCR in urine samples showed the presence of BKPyV with OPQRS as well as with rearranged structure. OPQRS was the predominant type of NCCR in urine samples collected at T1 and T2. Comparison of NCCR sequences per patient showed no differences (100% identity) in most cases (n = 22, 81.5%). Changes were observed in urine samples for 5 patients (18.5%) (Fig. 4); none of these patients were treated with cidofovir. OPQRS NCCR was detected in plasma samples collected at time points 1 (n = 8) and 2 (n = 5), and rearranged NCCR was only detected at time point 2 (n = 3). Changes were detected in plasma samples for 4 patients (50%), including one patient (Patient 2) treated with cidofovir (Fig. 4).

#### Amino acid changes in BKPyV protein-coding region were found in one third of paired urine and plasma samples

**Figure 5:**
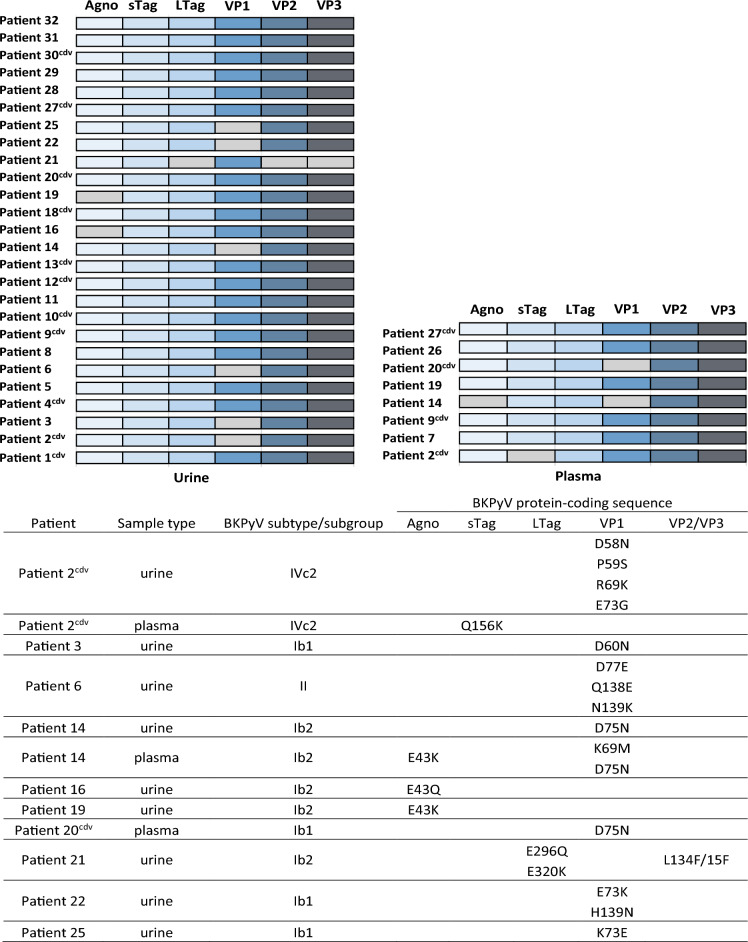
Upper panel: visual representation of genetic changes observed in BKPyV Agno, sTag, LTag, VP1, VP2, and VP3—protein coding sequences from urine and plasma samples collected at T1 and T2 in 26 and 8 kidney recipients, respectively. Lower panel: overview of patients who had amino acid changes in urine and/or plasma samples. Each bar shows BKPyV-protein coding sequences obtained at T1 and T2 for one patient. Light gray indicates that the sequences were different at both T1 and T2 for the patient. cdv, cidofovir.

Comparison of the BKPyV-protein coding sequences obtained in urine samples at T1 and T2 per patient showed that they were identical (100%) in most cases (n = 17; 65.4%). Nucleotide changes were found in urine samples from 9 patients (34.6%), including one patient (Patient 2) treated with cidofovir (see Supplementary Table S3). The identified nucleotide changes were associated with amino acid substitutions in Agno (n = 2; 22.2%), LTag (n = 1; 11.1%), VP1 (n = 6; 66.6%), and VP2/VP3 (n = 1; 11.1%) (Fig. 5). Comparison of plasma samples collected at T1 and T2 per patient showed nucleotide changes in BKPyV Agno, sTag, LTag, and VP1 protein-coding sequences in 4 of 8 patients. No differences were observed for other 4 patients (see Supplementary Table S4). Amino acid changes were found in plasma samples from 3 patients (37.5%), including 2 patients (Patient 2 and Patient 20) treated with cidofovir. Changes were detected in Agno (n = 1; 33.3%), in sTag (n = 1; 33.3%), and in VP1 (n = 2; 66.6%) (Fig. 5).

#### Changes in BKPyV VP1 protein consisted in the introduction of a previously unseen amino acid variant at positions 75 and 139 in subgroup Ib1, at positions 69 and 75 in Ib2 and at positions 58, 59, and 73 in IVc2

In Table 1, we listed the amino acid variants observed in proteins of BKPyV subtypes/subgroups Ib1, Ib2, II and IVc2 at positions where changes were found based on the analysis of 34 T2 samples (26 urine and 8 plasma samples). We compared them with the amino acid variants identified in the same BKPyV subtypes/subgroups at the same positions from three-month samples (total 126 samples, including 34 T1 samples)^35^. The analysis showed that VP1 amino acid variants identified at T2 in Ib1 at positions 60 and 73 (60N and 73K) and in IVc2 at position 69 (69K) were also present in samples at T1. In addition, there were amino acid variants that were found only in samples at T2. Previously unseen variants have been found at positions 75 and 139 (75N and 139N) in Ib1 and at positions 58, 59, and 73 (58N, 59S and 73G) in IVc2. In IVc2, the amino acid variant at position 156 (156K) in LTag was found only in samples at T2. In subgroup Ib2, the amino acid variants at position 43 (43Q) in Agno, at 296 and 320 (296Q and 320K) in LTag, at 69 and 75 (69M and 75N) in VP1, and at 134/15 (134/15F) in VP2/VP3 were only detected in samples at T2. An amino acid variant (K) at the same position in Agno (43), was also found in samples at T1. In subtype II, amino acid variants at positions 77 and 139 (77E and 139K) were found only in samples at T2. It has to be noted, however, that the results for subtype II must be treated with caution as the analysis was based only on 5 T1 and one T2 samples (Table 1).

**Table 1:** Amino acid variants identified in six BKPyV proteins in samples collected at T1 and T2 from kidney transplant recipients.

BKPyV subtype/subgroup	T1, sample (*n* = 126)	T2, sample (*n* = 34)	Amino acid variant														
			Agno			sTag			LTag			VP1			VP2/VP3		
			Position	T1 (*n*)	T2 (*n*)	Position	T1 (*n*)	T2 (*n*)	Position	T1 (*n*)	T2 (*n*)	Position	T1 (*n*)	T2 (*n*)	Position	T1 (*n*)	T2 (*n*)
Ib1	21	5										60	D (20)	D (4)			
													N (1)	N (1)			
												73	E (20)	E (4)			
													K (1)	K (1)			
												75	D (21)	D (4)			
													N (0)	N (1)			
												139	H (21)	H (4)			
													N (0)	N (1)			
Ib2	76	20	43	E (75)	E (18)				296	E (76)	E (19)	69	K (76)	K (19)	134/15	L (76)	L (19)
				K (1)	K (2)					Q (0)	Q (1)		M (0)	M (1)		F (0)	F (1)
			43	E (76)	E (19)				320	E (76)	E (19)	75	D (76)	D (18)			
				Q (0)	Q (1)					K (0)	K (1)		N (0)	N (2)			
IVc2	24	8				156	Q (24)	Q (7)				58	D (24)	D (7)			
							K (0)	K (1)					N (0)	N (1)			
												59	P (24)	P (7)			
													S (0)	S (1)			
												69	R (23)	R (7)			
													K (1)	K (1)			
												73	E (24)	E (7)			
													G (0)	G (1)			
II	5	1										77	D (5)	D (0)			
													E (0)	E (1)			
												138	E (4)	E (1)			
													Q (1)	Q (0)			
												139	N (5)	N (0)			
													K (0)	K (1)			

#### Genetic changes in JCPyV were found in one paired urine sample

Per-patient comparison showed no changes in either the genotype/subtype or the NCCR structure for any of patients. Analysis of the rest of the JCPyV genome revealed nucleotide changes in samples from one patient (see Supplementary Table S5, Fig. S1 and S2). The identified nucleotide changes were associated with amino acid substitutions in the VP1 protein at position 52 (aspartic acid (D) vs. asparagine (N)) and 70 (glycine (G) vs. serine (S)).

## Discussion

We characterized BKPyV genome in urine and plasma samples collected from kidney transplant recipients with persisting viremia over time post-transplant. Per-patient comparison of previously obtained viral sequences in the first months after transplantation with those obtained in later samples allowed us to identify viral genomic changes occurring over time.

The subtype/subgroup of the BKPyV detected in the patients remained mostly unchanged between the first and later time-point after transplantation. We showed previously that the donor kidney was the main source of replicating BKPyV subtype/subgroup in kidney transplant recipients early after transplantation^35^. Detection of the same BKPyV subtype/subgroup in the same patient much later after transplantation indicates the predominance of the donor-derived viral subtype/subgroup in kidney recipients also at later stages. There was one patient who had two different BKPyV subgroups at two time-points. It can be assumed that the new BKPyV subgroup appeared either as a result of a re-infection or due to the reactivation of a pre-existent BKPyV in the recipient. We also cannot completely rule out the possibility of human error in sampling.

BKPyV with archetypal (OPQRS) NCCR structure was the predominant genetic variant found in urine samples collected at both time-points. Comparison of NCCR sequences in urine per patient showed that they were 100% identical in most cases. OPQRS was also the main pattern of BKPyV NCCR in plasma samples collected at first time-point after transplantation. Comparison of sequences in plasma per patient showed in half of the cases changes in the BKPyV NCCR. In line with previous studies about the emergence of BKPyV with rearranged NCCR in kidney transplant recipients^20^, we have also observed an increase in plasma viral load in our cohort’s patients with changes in BKPyV NCCR. We, however, cannot draw any firm conclusions about the clinical significance of the increase, given the limited number of BKPyV DNA sequences obtained from plasma samples.

Amino acid changes were detected in all six BKPyV proteins of our sample collection: changes in the VP1 and Agno proteins were detected in both urine and plasma samples, changes in LTag, VP2 and VP3—in urine samples, and changes in sTag—in plasma samples. With regard to the BKPyV subtype/subgroup, VP1 changes were found in Ib1, Ib2, IVc2 and II, VP2/VP3, Agno, and LTag changes—only in Ib2 and sTag changes—in IVc2.

Amino acid changes in the VP1 protein were found at positions 58, 59, 60, 69, 73, 75, 77, 138 and 139, which corresponds to the two external protein loops, BC (amino acid range 57–89) and HI (127–146)^31^. These observations are in line with previous studies showing accumulation of mutations over time in the BKPyV VP1 outer loops from kidney transplant recipients^33,34,43^. Amino acid changes were identified at positions 60, 69, 73, 75, and 139 in subgroups Ib1 and Ib2. The BC-loop amino acid substitutions at positions 69 and 75 (K69M and D75N) were found only in samples collected later after kidney transplantation. The amino acid changes at positions 60 and 73 (D60N and E73K) were also observed in earlier patient samples. Worth mentioning is that at position 73, we have also observed a reversion to the conserved amino acid (K73E), supporting the previously proposed hypothesis about the existence of sites susceptible to reverse toggling in BKPyV VP1 BC-loop^33^. In subgroup IVc2, the amino acid changes were found at positions 58, 59, 69, and 73. The amino acids substitutions at three of these positions (D58N, P59S, and E73G) were only identified in later post-transplant samples while the amino acid change at position 69 (R69K) was also found in earlier post-transplant samples. Comparable results were obtained in a recent study analyzing 45 VP1 sequences from kidney transplant recipients. The analysis of the BC-loop showed that the most frequently mutated amino acids in subtype I and IV were located at positions 60, 72, 73, 75, and 82 and at positions 62, 69, 73, and 77, respectively. In vitro assays showed that the virus with BC-loop variants (in particular Ib2 with D60N, K69N, A72V, and E82Q and IVc2 with N61S, R69K, E73A, and D77N) were able to escape neutralization from patient serum^34^. It has been suggested that the accumulation of VP1 neutralizing antibody escape mutations over time could have implications for therapies involving monoclonal antibodies or intravenous immunoglobulins in kidney transplant recipients. It has been proposed that such therapies may be more effective if used as a pre-emptive rather than as a curative approach^34^. In addition, Peretti et al. have recently shown promising vaccination results on rhesus macaques using the virus-like particles (VLPs) approach^44^. Monkeys immunized with a mixture of different BKPyV subtypes/subgroups (Ib2, II, IVc2) plus one JCPyV genotype were able to develop a high and sustained neutralizing antibody response. In addition, primate sera demonstrated neutralizing capacity against BKPyV variants with VP1 BC-loop mutations, implying that a multivalent polyomavirus vaccine could be used as a preventive strategy in kidney transplant recipients to better control post-transplant BKPyV replication^44^.

Amino acid changes in the Agno protein were found in three different samples, whereas changes in sTag, LTag, VP2/VP3 proteins—in only one sample. Amino acid changes in sTag, LTag and VP2/VP3 were only found in samples collected later after transplantation while, amino acid changes in Agno were also found in earlier samples.

The change in the Agno protein occurred at the same position (43) in all three samples. The Agno protein has so far only been found in a few polyomaviruses, including JCPyV and Simian virus 40 (SV40)^7^. Comparison of the Agno protein sequences showed that amino acid at position 43 is located in a short-conserved region of the protein in all three different polyomaviruses (see Supplementary Fig. S3). It has recently been shown that the central part of the Agno protein (amino acids from 20 to 42) forms an amphipathic helix and is required for targeting lipid droplets in BK infected primary human renal tubular cells^45^. In addition, it has been shown that the Agno protein co-localizes with mitochondria and that the amphipathic central part is necessary for the disruption of mitochondria^46^. It can be assumed that the close proximity of the identified amino acid change site to this central region may have the implications as to the interaction with cellular comportments. Further work is warranted to better understand the role of the identified mutations in the lifecycle and pathogenesis of BKPyV in kidney transplant recipients.

Cidofovir is an analogue of deoxycytidine monophosphate used to treat infections caused by different DNA viruses^47^. In our patient cohort, approximately one-third of the patients (n = 12) received cidofovir for the treatment of the BKPyV infection. In those patients, we found genetic changes in both the non-coding and coding regions of the BKPyV genome in urine as well as plasma samples. The frequency of mutations was lower when compared to the patients who did not receive the drug. In vitro studies showed that cidofovir can inhibit BKPyV replication in human renal tubular cells^48^. It can be assumed that the less the virus replicates, the less chances there are for it to change. Similarly, the low frequency of mutation observed in samples from patients treated with cidofovir may indicate inhibition (at least temporarily) of viral replication. Along with cidofovir, the treatment was accompanied with the cessation of mycophenolate-mofetil treatment in the patients. Therefore, it is unclear whether the results obtained are due to cidofovir alone or to changes in immunosuppression.

Comparison of the JCPyV genome sequence in urine samples collected at two time-points showed nucleotide changes in the JCPyV VP1 protein. Identified changes were associated with amino acid substitutions at position 52 and 70 in the JCPyV VP1 BC outer loop. It was previously shown that substitutions in the VP1 protein can affect the cell tropism of JCPyV, favoring attachment to brain glial cells and the development of progressive multifocal leukoencephalopathy (PML)^49^. Although none of the VP1 mutations identified in this work matched previously recognized neurotropic JCPyV VP1 mutations^49^, our work confirms the occurrence of mutations in the JCPyV VP1 after kidney transplantation^5^.

In conclusion, our study showed that changes can occur over time in both the non-coding control region and the entire coding region of the BKPyV genome after kidney transplantation. Amino acid changes in VP1 were more frequent as compared to other BKPyV proteins and mainly affected the protein outer BC-loop. The identified BC-loop amino acid changes included those previously shown to confer resistance to neutralization. The number of BC-loop amino acid changes was higher in later samples as compared to those collected earlier after transplantation, implying that antibody-based therapy such as intravenous immunoglobulins preparation is best applied in the early stages of viral replication in kidney transplant recipients.

### Supplementary Information

Supplementary Information.

## Data Availability

The datasets generated and/or analyzed during the current study are available in GenBank under accession numbers OQ230834–OQ230874 (BK polyomavirus genome sequences) and OQ230875–OQ230886 (JC polyomavirus genome sequences).
