# Supplementary material for: Time-dependent variations in BK polyomavirus genome from kidney transplant recipients with persistent viremia
Source: Sci Rep. 2023 Aug 19;13:13534. doi: 10.1038/s41598-023-40714-4 (PMC10439958; doi:10.1038/s41598-023-40714-4)
Supplement: Supplementary file 1 — Supplementary Information. [file 41598_2023_40714_MOESM1_ESM.pdf]

Time-dependent variations in BK polyomavirus genome from kidney transplant recipients with persistent viremia.

Mineeva-Sangwo Olga, Van Loon Elisabet, Andrei Graciela, Kuypers Dirk, Naesens Maarten and Snoeck Robert

Table S1. Primer sequences

Target	Primer		Sequence (5' - 3')
BKPyV genome	Pair 1	Forward	CTATTGCTGGGTTTGCTGCT
		Reverse	AGAACCCAAATATTTCCACCAGG
	Pair 2	Forward	CAGTGCTTGATCCATGTCCAG
		Reverse	GGCATTTACAATTGTCCAGGTAG
JCPyV genome	Pair 1	Forward	GTAGCTGGGTTTGCTGCATTGG
		Reverse	ACAGGTGTTTCCACCTGGAATT
	Pair 2	Forward	CCATGTCCAGAGTCTTCTGCT
		Reverse	TGGTGAATTAACTATTGCCCAAG
JCPyV NCCR		Forward	GGAGGAAAATCACAAC
		Reverse	CGGCACCCATGAACCT

Abbreviations: BKPyV, BK polyomavirus; JCPyV, JC polyomavirus; NCCR, non-coding control region.

Table S2. Sequences used for JCPyV genotyping

GenBank access number	JCPyV genotype/subtype
AF015526.1	1/1A
AF015527.1	1/1B
AF015530.1	2/2A
AF015533.1	2/2B
AF015535	2/2C
U73502.1	3/3A
U73501.1	3/3B
AF015528.1	genotype 4
AF015537.1	genotype 6
U61771.1	genotype 7
AF015536.1	subtype 2D1
AF281605.1	subtype 2E
AF300961	subtype 7A
AF300946	subtype 7B1
AF300959	subtype 7C1
AF281626	subtype 8A
AF281624	subtype 8B

Table S3. BKPyV nucleotide changes identified in urine samples collected at two different post-transplant time points from 9 kidney recipients

Patient	BK polyomavirus protein-coding sequence					
	Agno	sTag	LTag	VP1	VP2	VP3
Patient 2[cdv]			1320 G>T	172G>A<br>175C>T<br>206G>A<br>218A>G		
Patient 3				178G>A		
Patient 6				231C>G<br>412C>G<br>417T>A		
Patient 14				223G>A		
Patient 16	127G>C	39C>T	39C>T			
Patient 19	127G>A					
Patient 21			886 G>C<br>958 G>A		402G>T	45G>T
Patient 22				217G>A<br>415C>A		
Patient 25				217 A>G		

Table S4. BKPyV nucleotide changes identified in plasma samples collected at two different post-transplant time points from 4 kidney recipients

Patient	BK polyomavirus protein-coding sequence					
	Agno	sTag	LTag	VP1	VP2	VP3
Patient 2[cdv]		466C>A				
Patient 14	127G>A			206A>T<br>223G>A		
Patient 19		99C>G	99C>G			
Patient 20[cdv]				223G>A		

Table S5. Characteristics of JCPyV sequences obtained in urine samples collected at T1 and T2 from kidney transplant recipients

Patient	Time-point	Genotype /Subtype	NCCR structure	Genome sequence identity*, % (bp/bp)
Patient 1	T1	4	ABCDEF	100 (4842/4842)
	T2	4	ABCDEF	
Patient 12	T1	N/A	N/A	N/A
	T2	4	ABCDEF	
Patient 13	T1	N/A	N/A	N/A
	T2	4	ABCDEF	
Patient 18	T1	4	ABCDEF	99.9 (4840/4842)
	T2	4	ABCDEF	
Patient 20	T1	2b	ABC(D)EF	100 (4841/4841)
	T2	2b	ABC(D)EF	
Patient 23	T1	N/A	N/A	N/A
	T2	4	ABCDEF	
Patient 25	T1	4	ABCDEF	N/A
	T2	N/A	N/A	
Patient 27	T1	1b	ABCDEF	N/A
	T2	N/A	N/A	
Patient 30	T1	2b	ABCDE(F)	N/A
	T2	N/A	N/A	

*JCPyV genome sequence minus non-coding regulatory region starting from the nucleotide at position 5118 to the nucleotide at position 276 (numbering follows that of NC_001699.1). Parentheses indicate a truncated NCCR block. Abbreviations: bp, base pairs; N/A, not available.

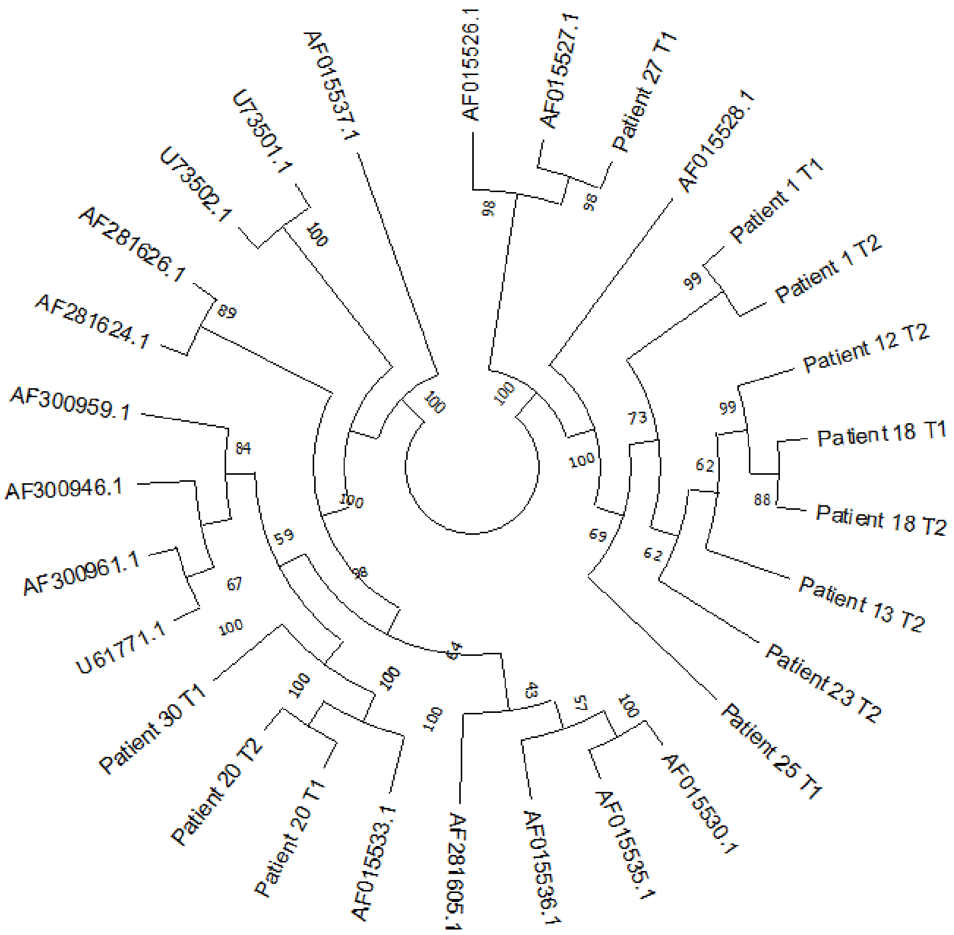

Figure S1. Maximum likelihood phylogenetic tree of JCPyV.

 1. M35834.1
 2. Patient 1_T1
 3. Patient 1_T2
 4. Patient 12_T2
 5. Patient 13_T2
 6. Patient 18_T1
 7. Patient 18_T2
 8. Patient 20_T1
 9. Patient 20_T2
10. Patient 23_T2
11. Patient 25_T1
12. Patient 27_T1
13. Patient 30_T1

```
    1            A                               B                                       C
    .........+.........+.........+.........+.........+.........+.........+.........+.........+.........+.........+.........+
    oooooooooooooooooooooooooooooooooooooooooooooooooooooooooooooooooooooooooooooooooooooooooooooooooooooooooooooooooooooooo
 1  gcctcggcctcctgtatatataaaaaaaagggaaggtagggaggagctggctaaaactggatggctgccagccaagcatgagctcatacctagggagccaaccagctgacagccagaggg
 2  gcctcggcctcctgtatatataaaaaaaagggaaggtagggaggagctggctaaaactggatggctgccagccaagcatgagctcatacctagggagccaaccagctgacagccagaggg
 3  gcctcggcctcctgtatatataaaaaaaagggaaggtagggaggagctggctaaaactggatggctgccagccaagcatgagctcatacctagggagccaaccagctgacagccagaggg
 4  gcctcggcctcctgtatatataaaaaaaagggaaggtagggaggagctggctaaaactggatggctgccagccaagcatgagctcatacctagggagccaaccagctgacagccagaggg
 5  gcctcggcctcctgtatatataaaaaaaagggaaggtagggaggagctggctaaaactggatggctgccagccaagcatgagctcatacctagggagccaaccagctgacagccagaggg
 6  gcctcggcctcctgtatatataaaaaaaagggaaggtagggaggagctggctaaaactggatggctgccagccaagcatgagctcatacctagggagccaaccagctgacagccagaggg
 7  gcctcggcctcctgtatatataaaaaaaagggaaggtagggaggagctggctaaaactggatggctgccagccaagcatgagctcatacctagggagccaaccagctgacagccagaggg
 8  gcctcggcctcctgtatatataaaaaaaagggaaggtagggaggagctggctaaaactggatggctgccagccaagcatgagctcatacctagggagccaaccagctgacagccagaggg
 9  gcctcggcctcctgtatatataaaaaaaagggaaggtagggaggagctggctaaaactggatggctgccagccaagcatgagctcatacctagggagccaaccagctgacagccagaggg
10  gcctcggcctcctgtatatataaaaaaaagggaaggtagggaggagctggctaaaactggatggctgccagccaagcatgagctcatacctagggagccaaccagctgacagccagaggg
11  gcctcggcctcctgtatatataaaaaaaagggaaggtagggaggagctggctaaaactggatggctgccagccaagcatgagctcatacctagggagccaaccagctgacagccagaggg
12  gcctcggcctcctgtatatataaaaaaaagggaaggtagggaggagctggctaaaactggatggctgccagccaagcatgagctcatacctagggagccaaccagctgacagccagaggg
13  gcctcggcctcctgtatatataaaaaaaagggaaggtagggaggagctggctaaaactggctggctgccagccaagcatgagctcatacctagggagccaaccagctgacagccagaggg
    ************************************************************ ***********************************************************

    121                     D                                       E                                             F
    .........+.........+.........+.........+.........+.........+.........+.........+.........+.........+.........+.........+
    ooooooooooooooooooooooooooooo              ooooooooooooooooooooooooooooooooooooooooooooooooooooooooooooooooooooo
    agccctggctgcatgccactggcagttatagtgaaacccctcccatagtccttaatcacaagtaaacaaagcacaaggggaagtggaaagcagccaggggaacatgttttgcgagccaga
    agccctggctgcatgccactggcagttatagtgaaacccctcccatagtccttaatcacaagtaaacaaagcacaaggggaagtggaaagcagccaagggaacatgttttgcgagccaga
    agccctggctgcatgccactggcagttatagtgaaacccctcccatagtccttaatcacaagtaaacaaagcacaaggggaagtggaaagcagccaagggaacatgttttgcgagccaga
    agccctggctgcatgccactggcagttatagtgaaacccctcccatagtccttaatcacaagtaaacaaagcacaaggggaagtggaaagcagccaagggaacatgttttgcgagccaga
```

```
agccctggctgcatgccactggcagttatagtgaaacccctcccatagtccttaatcacaagtaaacaaagcacaaggggaagtggaaagcagccaagggaacatgttttgcgagccaga
agccctggctgcatgccactggcagttatagtgaaacccctcccatagtccttaatcacaagtaaacaaagcacaaggggaagtggaaagcagccaagggaacatgttttgcgagccaga
agccctggctgcatgccactggcagttatagtgaaacccctcccatagtccttaatcacaagtaaacaaagcacaaggggaagtggaaagcagccaagggaacatgttttgcgagccaga
agccctggctgcatgccactggcagttat--------------catagtccttaatcacaagtaaacaaagcacaaggggaagtggaaagcagccaagggaacatgttttgcgagccaga
agccctggctgcatgccactggcagttat--------------catagtccttaatcacaagtaaacaaagcacaaggggaagtggaaagcagccaagggaacatgttttgcgagccaga
agccctggctgcatgccactggcagttatagtgaaacccctcccatagtccttaatcacaagtaaacaaagcacaaggggaagtggaaagcagccaagggaacatgttttgcgagccaga
agccctggctgcatgccactggcagttatagtgaaacccctcccatagtccttaatcacaagtaaacaaagcacaaggggaagtggaaagcagccaagggaacatgttttgcgagccaga
agccctggctgcatgccactggcagttatagtgaaacccctcccatagtccttaatcacaagtaaacaaagcacaaggggaagtggaaagcagccaagggaacatgttttgcgagccaga
agccctggctgcatgccactggcagttatagtgaaacccctc-cctagtccttaatcacaagtaaacaaagcacaagggaagtggaaagcagccaagggaacatgttttgc--------
*****************************              * ***************************************************o***************

241
.........+.........+.........+
             ooooooooooooooooo
gctgttttggcttgtcaccagctggccatg
gctgttttggcttgtcaccagctggccatg
gctgttttggcttgtcaccagctggccatg
gctgttttggcttgtcaccagctggccatg
gctgttttggcttgtcaccagctggccatg
gctgttttggcttgtcaccagctggccatg
gctgttttggcttgtcaccagctggccatg
gctgttttggcttgtcaccagctggccatg
gctgttttggcttgtcaccagctggccatg
gctgttttggcttgtcaccagctggccatg
gctgttttggcttgtcaccagctggccatg
gctgttttggcttgtcaccagctggccatg
-------------gtcaccagctggccatg
             *****************
```

Figure S2. Comparison of JCPyV NCCR in urine samples collected at post-transplant time points 1 (T1) and 2 (T2) from 9 kidney recipients.

```
CLUSTAL Omega(1.2.4) multiple sequence alignment

P03084 - Simian virus 40
P03086 - JC polyomavirus
P03085 - BK polyomavirus
Q3L6L9 - Simian virus 12

sp|P03084|AGNO_SV40       MVLRRLSRQASVKVRRSWTESKKTAQRLFVFVLELLLQFCEGEDTVDGKRKKPERLTEKP    60
sp|P03086|AGNO_POVJC      MVLRQLSRKASVKVSKTWSGTKKRAQRILIFLLEFLLDFCTGEDSVDGKKRQRHSGLTEQ    60
sp|P03085|AGNO_POVBK      MVLRQLSRQASVKVGKTWTGTKKRAQRIFIFILELLLEFCRGEDSVDGKNKSTTALPAVK    60
sp|Q3L6L9|AGNO_POVS1      MVLRQLSRQASVKVGKTWTGTKRRAQRIFIFILELLLDFCRGEDSVDGKKKKDSLTDKTE    60
                          ****:***:***** ::*: :*: ***:::*:**:**:** ***:****.:.

sp|P03084|AGNO_SV40       E------S---   62
sp|P03086|AGNO_POVJC      TYSALPEPKAT   71
sp|P03085|AGNO_POVBK      DSVKDS-----   66
sp|Q3L6L9|AGNO_POVS1      TVTEKKES---   68
```

Figure S3. Comparison of Agno amino acid sequences of BKPyV (P03085), JCPyV (P03086 and Q3L6L9) and SV40 (P03084).
